# Six minutes to promote change: People, not facts, alter students' perceptions on climate change

**DOI:** 10.1002/ece3.7553

**Published:** 2021-05-01

**Authors:** Kodiak A. Sauer, Daniel K. Capps, David F. Jackson, Krista A. Capps

**Affiliations:** ^1^ Department of Psychology University of Georgia Athens GA USA; ^2^ Department of Mathematics and Science Education University of Georgia Athens GA USA; ^3^ Odum School of Ecology University of Georgia Athens GA USA; ^4^ The Savannah River Ecology Laboratory University of Georgia Aiken SC USA

**Keywords:** climate change, cultural cognition, gateway belief model, knowledge deficit model, moral foundations theory, pedagogy, socioscientific issues

## Abstract

Anthropogenic climate change threatens the structure and function of ecosystems throughout the globe, but many people are still skeptical of its existence. Traditional “knowledge deficit model” thinking has suggested that providing the public with more facts about climate change will assuage skepticism. However, presenting evidence contrary to prior beliefs can have the opposite effect and result in a strengthening of previously held beliefs, a phenomenon known as biased assimilation or a backfire effect. Given this, strategies for effectively communicating about socioscientific issues that are politically controversial need to be thoroughly investigated. We randomly assigned 184 undergraduates from an environmental science class to one of three experimental conditions in which we exposed them to short videos that employed different messaging strategies: (a) an engaging science lecture, (b) consensus messaging, and (c) elite cues. We measured changes in student perceptions of climate change across five constructs (content knowledge, acceptance of scientific consensus, perceived risk, support for action, and climate identity) before and after viewing videos. Consensus messaging outperformed the other two conditions in increasing student acceptance of the scientific consensus, perceived risk of climate change, and climate identity, suggesting this may be an effective strategy for communicating the gravity of anthropogenic climate change. Elite cues outperformed the engaging science lecture condition in increasing student support for action on climate, with politically conservative students driving this relationship, suggesting that the messenger is more important than the message if changing opinions about the necessity of action on climate change is the desired outcome. Relative to the other conditions, the engaging science lecture did not support change in students' perceptions on climate, but appealing to student respect for authority produced positive results. Notably, we observed no decline in students' acceptance of climate science, indicating that none of the conditions induced a backfire effect.

## Graphical abstract

University educators are required to teach socially and politically controversial concepts to students from a wide range of political backgrounds. At the same time, university professors are increasingly more politically liberal and less religious than the general population. Therefore, it can be challenging for professors to effectively convey information about topics that are socially and politically controversial to diverse groups of students. This study sought to evaluate the effectiveness of video‐based teaching strategies to support student acceptance of global, anthropogenic climate change.

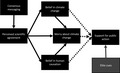

## 1 INTRODUCTION

Educators at all levels are required to teach socially controversial topics; yet, when students are presented with subjects that appear to contradict their beliefs, they may immediately reject the information, impeding meaningful learning. For instance, research in biology education has demonstrated that understanding evolution is foundational in learning biology (AAAS, 2011; Brownell et al., 2014). However, a student's religious beliefs can impede acceptance of evolution (Berkman & Plutzer, 2011; Pobiner, 2016). Personal beliefs can similarly impact one's willingness to accept the science behind vaccinations (Evrony & Caplan, 2017; Institute of Medicine, 2010; Nelson & Rogers, 1992) and genetically modified foods (Juma, 2011; National Academies of Sciences, Engineering, & Medicine. 2016; Potrykus, 2017). Studies have also demonstrated that the acceptance of anthropogenic climate change can be influenced by a student's personal beliefs (Hess & Maki, 2019; Wachholz et al., 2014).

Anthropogenic climate change threatens the integrity of social and ecological systems throughout the globe, and expert consensus suggests that dramatic action is necessary to avoid catastrophic environmental and socioeconomic outcomes (Cook et al., 2016; IPCC, 2018; Wolters & Steel, 2017). Presumably, action to mitigate continued climate change is made more likely if populations understand the risks. Hence, educators must critically evaluate the most effective ways to present this information to students. Traditionally, the perception that enhancing science content knowledge results in increased acceptance of the scientific consensus has governed the teaching practices of university educators (Sturgis & Allum, 2004; Simis et al., 2016; Kitta & Goldberg, 2017). Unfortunately, research has demonstrated that this model of teaching, which has been classified as the knowledge deficit model of scientific communication, is not closely correlated with perceived risk of climate change (Kahan et al., 2012).

Belief in politically and socially controversial topics can relate more to values held by an individual than to existing scientific evidence. In fact, presenting science without attention to personal values can result in a “backfire effect,” with people strengthening previously held beliefs (Cook & Lewandowsky, 2016; Hart & Nisbet, 2012; Nyhan & Reifler, 2010). For instance, political liberals may reject genetically modified foods, vaccines, or nuclear energy because they may be perceived as unnatural, stoking fear that human interaction will harm the environment (Dixon & Hubner, 2018). Similarly, individuals with certain religious affiliations may reject evolution because it is seen as counter to their belief system (Deniz & Borgerding, 2018; Glaze & Goldston, 2015; Mazur, 2004). Additionally, political conservatives may be skeptical of anthropogenic climate change due to the implications of government regulations that restrict behavior (Kahan et al., 2012). Collectively, this information indicates that belief in anthropogenic climate change can be inversely related to education and knowledge of climate science, and this may especially be true for politically conservatives (Hamilton, 2011).

Political identity can be a robust barrier to student acceptance of politically controversial scientific conclusions (Walker et al., 2017), and psychologists have tested different messaging strategies to reduce differences in environmental attitudes between political liberals and conservatives (Feinberg & Willer, 2013). For instance, moral foundations theory (Haidt & Graham, 2007, 2009; Haidt, 2008; Haidt, 2012; Graham et al., 2009; Graham et al., 2013) seeks to address the moral differences in liberals and conservatives in terms of a relatively simple dichotomy. On average, political liberals tend to care more about harm and fairness as they pertain to moral intuitions, whereas political conservatives tend to care more about in‐group loyalty, respect for authority, and purity or sanctity. It has been argued that these moral foundations are unlikely to be truly innate, modular, or neurobiologically descriptive (Suhler & Churchland, 2011). However, the theory can be a useful framework for describing and understanding people's motives and justifications, and the ways in which they are influenced by others, in an applied sociopolitical context (*inter alia,* Feinberg & Willer, 2013; Day et al., 2014; Low & Wui, 2016; Kalimeri et al., 2019; Christie et al., 2019). While some moral psychologists have questioned whether the latter three foundations of loyalty, authority, and purity (the “binding foundations” of Graham et al., 2009, 2011) are truly “moral” in an objective sense (Kugler et al., 2014), they are undoubtedly believed to be moral by large segments of human populations and are thus useful in a descriptive and pragmatist perspective of morality.

Problem‐solving to address climate change requires understanding, empathy, and respect across political divides. Therefore, educators should tailor lessons to effectively communicate to groups of students with diverse beliefs and different social and educational backgrounds (Moser & Dilling, 2011). Here, we applied insights from moral foundations theory to teaching climate change to university students. We tested the efficacy of three approaches, or conditions, to shift student perceptions on climate change in an introductory course for nonmajors. We developed short video conditions that presented information about climate change using (a) a specifically informative lecture by an expert scientist widely acknowledged as a highly engaging speaker, (b) a focused message on the scientific consensus surrounding climate change, or (c) elite cues about climate delivered by well‐known conservative politicians, military leaders, and other well‐established figures. The first two approaches were based on arguments commonly used to convince people of anthropogenic climate change: lecturing on the science (referred to as the engaging science lecture condition) and teaching the scientific consensus (referred to as the consensus messaging condition) of “97% of climate scientists agree on climate change” (Oreskes, 2004). The last condition, “elite cues,” reflects the respect for authority and in‐group loyalty that is associated with the moral foundations of more politically conservative people (*inter alia*, Haidt & Graham, 2007, 2009; Haidt, 2008; Haidt, 2012; Graham et al., 2009; Graham et al., 2013).

We expected that prior to exposure to the experimental conditions, students would vary in their climate beliefs in expected ways based on political ideology, with liberal students agreeing more with the scientific consensus than more politically conservative students. We also expected a political ideology by video condition interaction where liberal students would respond more effectively to the engaging science lecture, and conservative students would respond more effectively to the elite cues condition. Presenting the science of climate change without attention to personal values has sometimes resulted in a strengthening of previously held beliefs (Cook & Lewandowsky, 2016; Hart & Nisbet, 2012), as can exposure to scientific consensus without the consideration of the values of the target audience (Kahan et al., 2011). Therefore, we expected the elite cues condition would outperform the consensus messaging and engaging science lecture conditions in altering student opinion on climate change across all constructs. If acceptance of scientific consensus was also related to political affiliation (Kahan et al., 2011) and strongly influenced student responses, we expected the consensus messaging and the engaging science lecture conditions would result in contrary updating, with conservative students strengthening their rejection of climate science and liberal students strengthening their belief in the scientific consensus. Alternatively, if consensus messaging increased perceived scientific agreement for all students, and positively shifted their attitudes about climate, we expected that consensus messaging would positively affect all of the other climate constructs we considered (van der Linden et al., 2015).

## 2 METHODS

**FIGURE 1:**
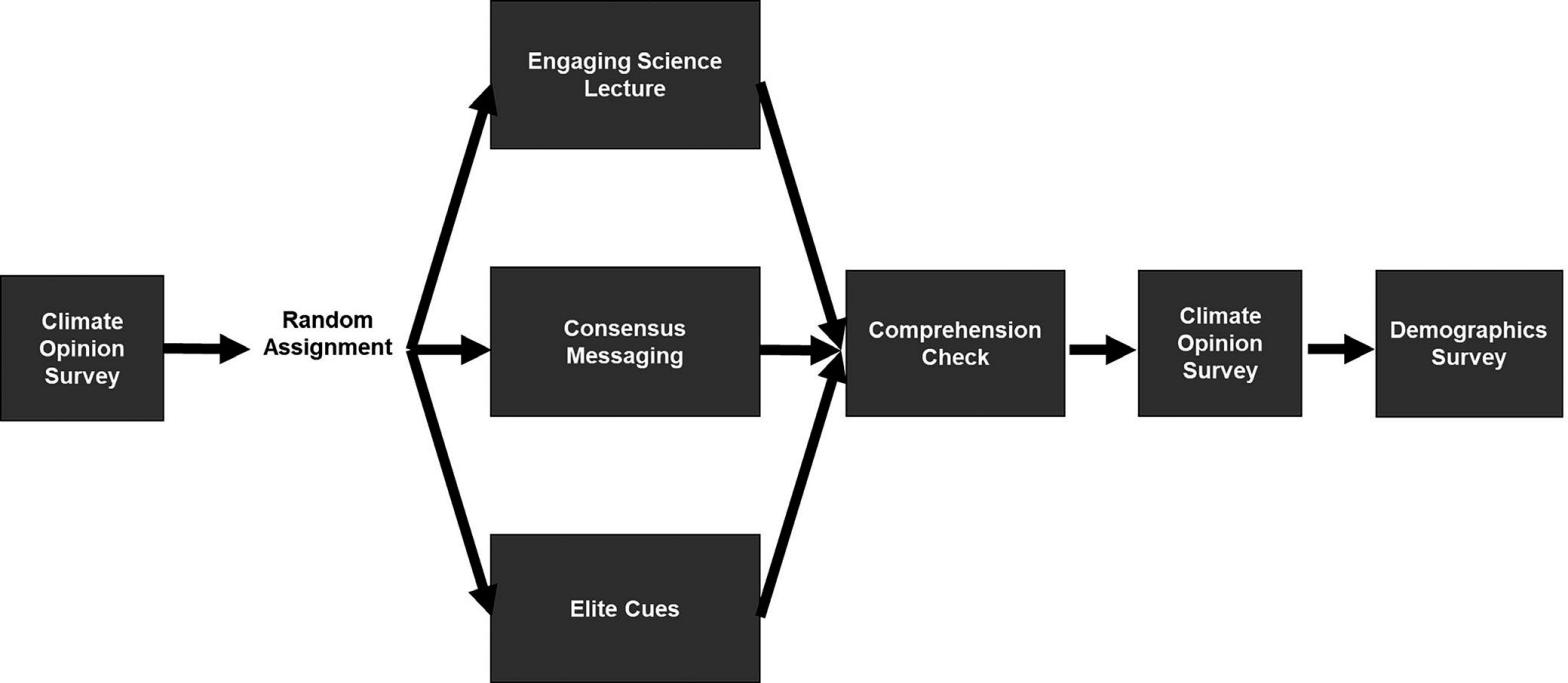
Study design. All students began with a uniform climate survey, were randomly assigned to a condition, completed a participation check, completed the same climate opinion survey, and then completed a uniform demographic survey

The study was conducted in an introductory‐level ecology and environmental science class at a large, public research institution in the southeastern United States. Since 1993, the university has had an Environmental Literacy requirement that states, “[students] must attain knowledge of basic principles concerning environmental issues.” We designed the study after methods described by Walker et al. (2017), which evaluated the determinants of student acceptance of politically controversial scientific conclusions (Figure 1). Focusing specifically on the topic of climate change, we extracted the six climate‐related statements from Walker and colleagues' survey. We grouped the statements into four conceptual categories: content knowledge, acceptance of scientific consensus, perceived risk, and climate identity. We then developed five additional statements so that we would have at least two items per category. We developed an additional two statements to evaluate a 5th category, as we were also interested in assessing student support for action to mitigate climate change (Table 2). Most statements were written as reversed pairs, in which we attempted to ask the same information in different ways to control for acquiescence (Winkler et al., 1982).

As part of the course, students were instructed to complete an online learning module designed to gauge their thoughts on climate change. They were informed that with their consent, their anonymized responses would be used as part of a research study and were given the option to opt out of the study with no penalty. They were then given a link to a Qualtrics (www.qualtrics.com) module and filled out a 13‐item, 5‐point Likert scale survey on their opinions on climate change. Upon completion of the survey, students viewed one of three 6‐min videos (see Appendix S1 to access survey questions, response data, and Videos S1, S2, S3 interventions) and completed a brief video comprehension check. They then completed the same 13‐item climate change opinion survey, followed by a brief demographic survey (Figure 1). Students were asked to fill out a demographic survey after they had completed the final climate opinion survey in an attempt to avoid priming students into group identifiers, something we thought could potentially influence their responses.

The key difference between the three conditions was the orientation of the 6‐min video on climate change (Figure 1). The video for the engaging science condition used excerpts of a Technology, Entertainment, and Design (TEDx) talk (Shepherd, 2013) by Dr. J. Marshall Shepherd, a well‐established Professor of Geography and climate scientist (e.g., President of the American Meteorological Society in 2013), who won the American Geophysical Union's 2019 Climate Communications Prize, to create an engaging science lecture focusing on the science content of anthropogenically produced climate change. We chose Dr. Shepherd's TEDx talk because we wanted to have a charismatic communicator give this condition its best chance of success. The video for the consensus messaging condition emphasized consensus within the scientific community on perceptions about anthropogenically mediated climate change. To develop the video, we integrated a small portion of Dr. Shepherd's TEDx talk, to introduce the “97% of climate scientists agree on climate change” statistic, with the Public Broadcasting Service's (PBS) “97% of Climate Scientists Really Do Agree” video (PBS Digital Studios, 2018). The video for the elite cues condition used an edited mixture of video clips of trusted conservative‐leaning political figures from the United States, religious figures, celebrities, and US military leaders talking about the urgency of climate change. All of the materials we used to create the video conditions were either in the public domain or labeled under a Creative Commons license.

### 2.1 Data analysis

Data were imported into IBM SPSS Statistics for Windows, Version 26.0, and were evaluated for quality prior to the analysis. Students who scored less than 67% on the comprehension check were eliminated from the analysis, as the comprehension check was designed to be simple enough that any student who paid attention to the video could easily answer the questions. Additionally, all student responses that were recorded within a duration that was shorter than the length of the video plus 30 s were eliminated, as this is the fastest we could reasonably expect a student to complete the study. We then plotted the data to see whether the expected trends in climate beliefs by political ideology held true in our sample. Next, we combined questions designed to assess the same construct into composite variables, and we evaluated the difference between pre‐ and post‐video scores for each participant. To assess the normality of data distribution, we plotted frequency histograms of the values. We then ran five separate two‐way univariate analyses of variance (ANOVA) tests on the difference scores, one for each of the climate constructs (content knowledge, acceptance of scientific consensus, perceived risk, support for action, climate identity), with experimental condition (engaging science lecture, consensus messaging, elite cues) and political ideology (liberal, middle of the road, conservative) as coequal factors in each ANOVA (Huberty & Morris, 1989). Post hoc Tukey's HSD tests were used to evaluate pairwise comparisons.

## 3 RESULTS

A total of 184 students of the 279 enrolled in the course chose to participate in the study. Of these, 80 (43.5%) identified themselves as male, 103 (56%) as female, and 1 (0.5%) as other. A total of 130 (70.7%) identified themselves as White/Caucasian, 26 (14.1%) as Asian/Pacific Islander, 21 (11.4%) as Black/African American, and 7 (3.8%) as Other. A total of 178 (96.7%) identified themselves as American citizens, and six students (3.3%) were foreign nationals. Fifty‐three students (28.8%) identified themselves as politically liberal, 67 (36.4%) as middle of the road politically, and 64 (34.8%) as politically conservative (Table 1).

**TABLE 1:** Participant demographic information

	Engaging science lecture		Consensus messaging		Elite cues		Full sample	
	*n*	%	*n*	%	*n*	%	*n*	%
Gender								
Male	27	46	28	41	25	44	80	43
Female	31	53	40	59	32	56	103	56
Other	1	2	0	0	0	0	1	1
Race								
Caucasian	35	59	53	78	42	74	130	71
African American	7	12	5	7	9	16	21	11
AAPI	13	22	7	10	6	11	26	14
Other	4	7	3	4	0	0	7	4
Political ideology								
Liberal	16	27	15	22	22	39	53	29
Middle of the road	25	42	26	38	16	28	67	36
Conservative	18	31	27	40	19	33	64	35
Total	59	32	68	37	57	31	184	100

Note

These data were collected from a survey that was completed after study participation to avoid priming students into group membership, something we thought could influence their study responses.

In prevideo survey responses, we documented the expected relationships between climate beliefs and political ideology (Table 2). Political ideology was predictive of climate change acceptance for most survey items. In contrast to our expectations, conservative students in our sample did not reject climate change. They agreed (mean response >3) with a majority of responses that affirmed the existence and gravity of anthropogenic climate change. Furthermore, in large part, they supported the consensus beliefs held by scientists (mean response >3). However, conservative students were also often uncertain in their responses, more often choosing the “neither agree nor disagree” option, especially when answering questions about consensus and identity.

**TABLE 2:** Mean prevideo survey responses from students

Group	Item	Political ideology			*F*	*p*
		Liberal	Middle of the road	Conservative		
Content knowledge	There is convincing evidence that human activities are altering the Earth's climate	4.74	4.27	3.95	9.939	.000
	Atmospheric concentration of carbon dioxide is increasing	4.32	3.84	3.91	5.123	.007
	Climate change has had an impact on recent extreme weather events	4.70	4.07	3.59	22.993	.000
	Human activities have too small of an impact to affect something as large as the Earth's climate	1.26	1.54	2.11	16.074	.000
Acceptance of consensus	Most scientists accept that human activities are altering the Earth's climate	4.47	4.04	4.27	3.827	.024
	Scientists are largely divided as to whether or not humans are the primary cause of climate change	2.19	2.55	3.19	14.422	.000
Perceived risk	Climate change is not a serious problem	1.09	1.55	2.13	24.379	.000
	Climate change poses a serious risk to human health, safety, and prosperity	4.94	4.39	4.00	23.238	.000
Support for action	Governments need to act to mitigate climate change	4.77	4.18	3.75	20.683	.000
	Individuals need to act to mitigate climate change	4.81	4.31	3.92	21.373	.000
Climate identity	People who accept that humans are causing climate change are very different from me	1.42	1.91	2.63	27.542	.000
	People who get worked up about climate change seem strange to me	1.49	2.10	2.98	42.805	.000
	I identify as a person who believes in climate change	4.85	4.18	3.67	29.165	.000

Note

Responses were categorized using student‐identified political beliefs collected in the survey. Results are mean values of 184 people with two degrees of freedom. Values greater than three indicate that the sample, on average, agreed with the statement more than they disagreed with it, and values <3 indicate that the sample disagreed with the statement more than they agreed. Thirteen one‐way ANOVAs revealed significant differences in Likert responses among students with varying political ideologies (5 = strongly agree, 4 = agree, 3 = neither agree nor disagree, 2 = disagree, 1 = strongly disagree).

**FIGURE 2:**
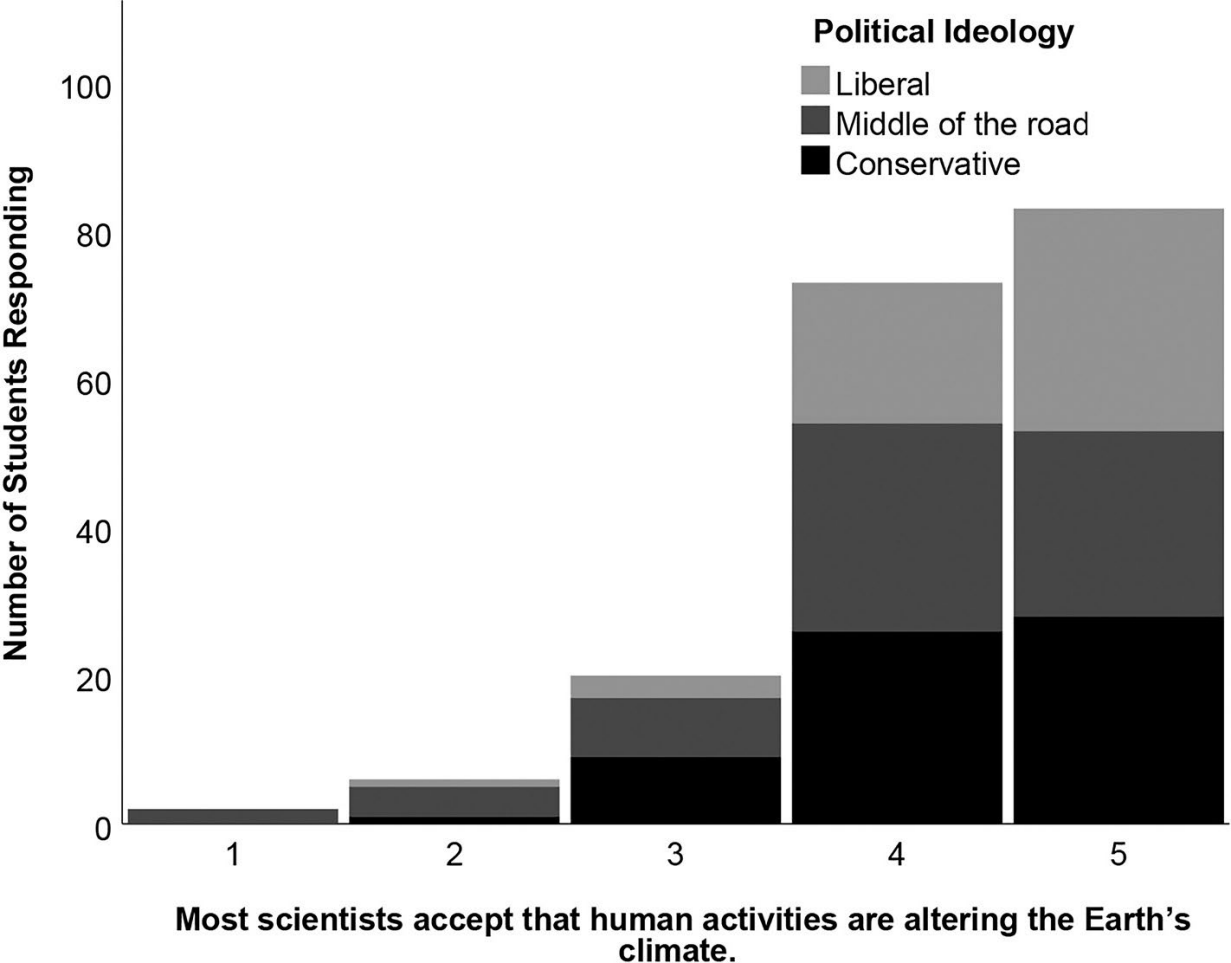
Histogram of student agreement with the statement, “Most scientists accept that human activities are altering the Earth's climate” prior to experimental interventions. Survey response data (5 = strongly agree, 4 = agree, 3 = neither agree nor disagree, 2 = disagree, 1 = strongly disagree). Color differences within the bars indicate variation in self‐identified political ideology

**FIGURE 3:**
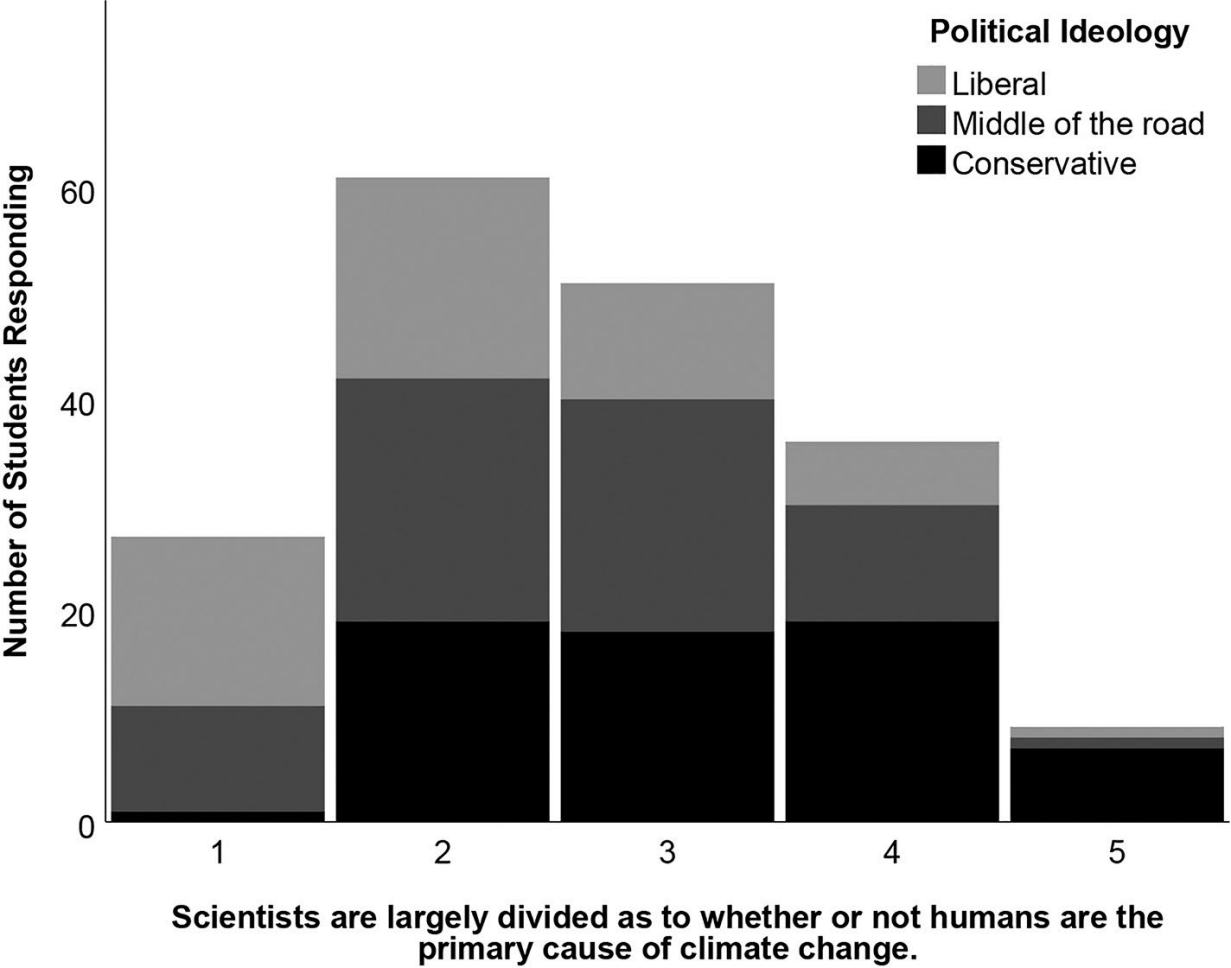
Histogram of student agreement with the statement, “Scientists are largely divided on whether or not humans are the primary cause of climate change” prior to experimental interventions. Survey response data (5 = strongly agree, 4 = agree, 3 = neither agree nor disagree, 2 = disagree, 1 = strongly disagree). Color differences within the bars indicate variation in self‐identified political ideology

Across all political ideologies, students were very likely to endorse the statement, “Most scientists accept that human activities are altering the Earth's climate” (Figure 2). Yet students that identified themselves as conservative were most likely to respond, “neither agree nor disagree,” to the statement, “Scientists are largely divided on whether or not humans are the primary cause of climate change,” prior to experimental intervention (Figure 3). Notably, we intended these statements to be very similar to one another, just worded in a reversed fashion. However, the results suggest that students derived different meanings from these statements. Many of the conservative students agreed with the assertion that most scientists agree that humans contribute to climate change (mean = 4.3), yet they were still ambivalent about the degree to which scientists are divided as to whether humans are the primary cause of this change (mean = 3.2).

**FIGURE 4:**
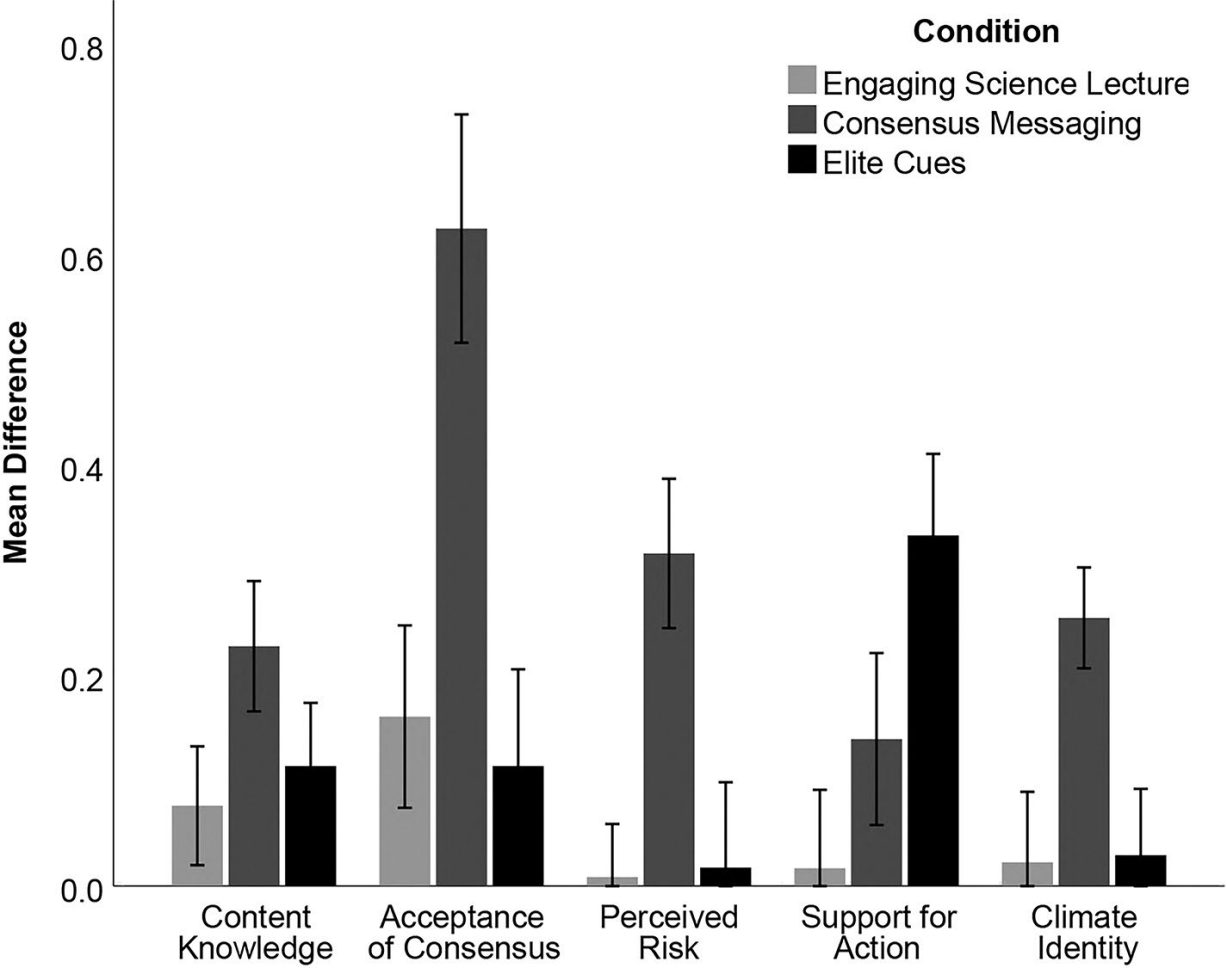
Mean difference scores between pre‐ and post‐video surveys among five assessed conceptual constructs relating to anthropogenic climate change for 184 students randomly assigned to three experimental conditions (error bars = ±1*SE*)

Experimental condition significantly affected the difference between pre‐ and post‐assessments in four of the five constructs (acceptance of scientific consensus (*p* < .001), perceived risk (*p* = .006), support for action (*p* = .017), climate identity (*p* = .025); Figure 4). However, experimental condition (i.e., the video a student watched) had no effect on their content knowledge (*p* = .134; content questions are detailed in Appendix S1). This was not surprising, as none of the three treatments were geared to provide detailed information about the impact of human activities on climate change. Political ideology did not significantly affect the difference in scores between pre‐ and post‐assessments in any of the constructs (content knowledge (*p* = .915), acceptance of scientific consensus (*p* = .839), perceived risk (*p* = .289), support for action (*p* = .706), climate identity (*p* = .384)). There were also no significant interactions between experimental condition and political ideology for any climate opinion construct (content knowledge (*p* = .898), acceptance of scientific consensus (*p* = .960), perceived risk (*p* = .774), support for action (*p* = .092), climate identity (*p* = .525); Table 3).

**TABLE 3:** Results from five separate two‐way univariate ANOVAs conducted on the difference scores for each of the climate constructs (content knowledge, acceptance of scientific consensus, perceived risk, support for action, climate identity)

	Sum of squares	*df*	Mean square	*F*	Sig.
Content knowledge					
Condition	0.921	2	0.461	2.035	0.134
Political ideology	0.040	2	0.020	0.089	0.915
Condition by political ideology	0.242	4	0.061	0.268	0.898
Total	44.50	184			
Acceptance of consensus					
Condition	10.173	2	5.087	8.415	***0.000***
Political ideology	0.212	2	0.106	0.176	0.839
Condition by political ideology	0.377	4	0.094	0.156	0.960
Total	135.25	184			
Perceived risk					
Condition	3.086	2	1.543	5.256	***0.006***
Political ideology	0.735	2	0.367	1.251	0.289
Condition by political ideology	0.526	4	0.131	0.448	0.774
Total	59.50	184			
Support for action					
Condition	3.087	2	1.544	4.146	***0.017***
Political ideology	0.260	2	0.130	0.349	0.706
Condition by political ideology	3.021	4	0.755	2.028	0.092
Total	76.25	184			
Climate identity					
Condition	1.617	2	0.808	3.779	***0.025***
Political ideology	0.412	2	0.206	0.962	0.384
Condition by political ideology	0.686	4	0.172	0.802	0.525
Total	43.00	184			

Note

Experimental condition (engaging science lecture, consensus messaging, elite cues) and political ideology (liberal, middle of the road, conservative) were considered as coequal factors in the analyses. Significant comparisons are denoted in bold, italicized font.

Post hoc comparisons using the Tukey HSD test (Table 4) revealed that the consensus messaging condition outperformed both the engaging science lecture and elite cues conditions at improving student acceptance of scientific consensus (*p* = .003, *p* = .001, respectively; Table 4A), perceived risk (*p* = .005, *p* = .007; Table 4B), and climate identity (*p* = .015, *p* = .020; Table 4D). In contrast, the only significant pairwise comparison for difference scores within the support for action construct was that the elite cues condition outperformed the engaging science lecture in increasing student support for action on climate (*p* = .016; Table 4C).

**TABLE 4:** Results from post hoc multiple comparisons using Tukey's HSD tests

(A) Dependent variable: acceptance of scientific consensus						
(I) Condition	(J) Condition	Mean difference (I‐J)	*SE*	Sig.	95% confidence interval	
					Lower bund	Upper bound
Engaging science lecture	Consensus messaging	−0.4640*	0.13833	***0.003***	−0.7910	−0.1370
	Elite cues	0.0470	0.14440	0.943	−0.2944	0.3883
Consensus messaging	Engaging science lecture	0.4640*	0.13833	***0.003***	0.1370	0.7910
	Elite cues	0.5110*	0.13962	***0.001***	0.1809	0.8410
Elite cues	Engaging science lecture	−0.0470	0.14440	0.943	−0.3883	0.2944
	Consensus messaging	−0.5110*	0.13962	***0.001***	−0.8410	−0.1809

(B) Dependent variable: perceived risk						
(I) Condition	(J) Condition	Mean difference (I‐J)	*SE*	Sig.	95% confidence interval	
					Lower bound	Upper bound
Engaging science lecture	Consensus messaging	−0.3077*	0.09640	***0.005***	−0.5356	−0.0798
	Elite cues	−0.0091	0.10063	0.996	−0.2469	0.2288
Consensus messaging	Engaging science lecture	0.3077*	0.09640	***0.005***	0.0798	0.5356
	Elite cues	0.2986*	0.09731	***0.007***	0.0686	0.5286
Elite cues	Engaging science lecture	0.0091	0.10063	0.996	−0.2288	0.2469
	Consensus messaging	−0.2986*	0.09731	***0.007***	−0.5286	−0.0686

(C) Dependent variable: support for action						
(I) Condition	(J) Condition	Mean difference (I‐J)	*SE*	Sig.	95% confidence interval	
					Lower bound	Upper bound
Engaging science lecture	Consensus messaging	−0.1228	0.10857	0.497	−0.3794	0.1339
	Elite cues	−0.3164*	0.11333	***0.016***	−0.5843	−0.0485
Consensus messaging	Engaging science lecture	0.1228	0.10857	0.497	−0.1339	0.3794
	Elite cues	−0.1936	0.10958	0.184	−0.4527	0.0654
Elite cues	Engaging science lecture	0.3164*	0.11333	***0.016***	0.0485	0.5843
	Consensus messaging	0.1936	0.10958	0.184	−0.0654	0.4527

(D) Dependent variable: climate identity						
(I) Condition	(J) Condition	Mean difference (I‐J)	*SE*	Sig.	95% confidence interval	
					Lower bound	Upper bound
Engaging science lecture	Consensus messaging	−0.2323*	0.08229	***0.015***	−0.4268	−0.0378
	Elite cues	−0.0066	0.08590	0.997	−0.2097	0.1964
Consensus messaging	Engaging science lecture	0.2323*	0.08229	***0.015***	0.0378	0.4268
	Elite cues	0.2257*	0.08306	***0.020***	0.0293	0.4220
Elite cues	Engaging science lecture	0.0066	0.08590	0.997	−0.1964	0.2097
	Consensus messaging	−0.2257*	0.08306	***0.020***	−0.4220	−0.0293

Note

Based on observed means.

(A) The error term is mean square (error) = 0.604.

(B) The error term is mean square (error) = 0.294.

(C) The error term is mean square (error) = 0.372.

(D) The error term is mean square (error) = 0.214.

* The mean difference is significant at the 0.05 level.

Given that there is debate in the literature as to whether consensus messaging can increase support for action on climate (Kahan, 2016; van der Linden et al., 2015, 2017), and that our initial analysis revealed that “support for action” was the only construct that did not follow the trend of consensus messaging outperforming the other two conditions, we examined this construct in a bit more detail. A one‐sample *t*‐test revealed that the difference scores for this construct did not significantly differ from 0 for students exposed to the consensus messaging condition (*p* = .092), signifying that we cannot document evidence indicating that consensus messaging increases student support for action on climate.

**FIGURE 5:**
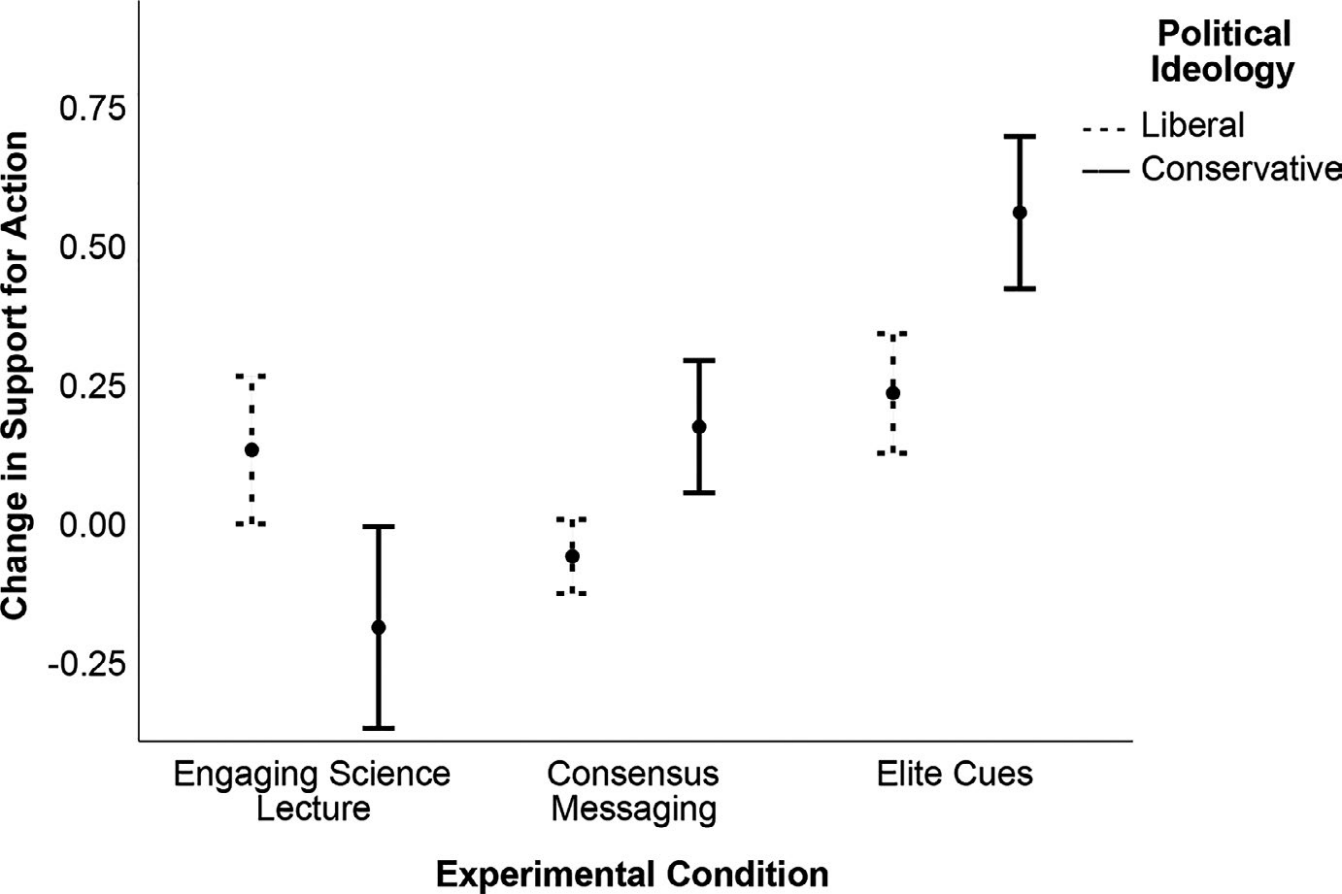
Mean change in support for action on climate change among 117 students self‐identifying as either liberal or conservative randomly assigned to three experimental conditions. The elite cues video effectively increased conservative student support for action on climate change (error bars = ±1*SE*)

We also wanted to evaluate whether our data indicated there was politically biased assimilation of information (Lord et al., 1979). We assumed that the “middle of the road” option represented students with more moderate political beliefs and reasoned that restricting the analysis to students that claimed polarized political ideologies might be more effective at capturing biased assimilation. Therefore, we ran an additional two‐way univariate ANOVA on the difference scores of this construct but restricted the analysis to two groups of political ideologies, liberal and conservative. This test produced the same main effect of experimental condition (*p* = .004), but not of political ideology (*p* = .466). We also documented a significant interaction between experimental condition and political ideology (*p* = .042; Figure 5), indicating elite cues may be the most effective strategy in enhancing conservative student's support for action on climate.

## 4 DISCUSSION

**FIGURE 6:**
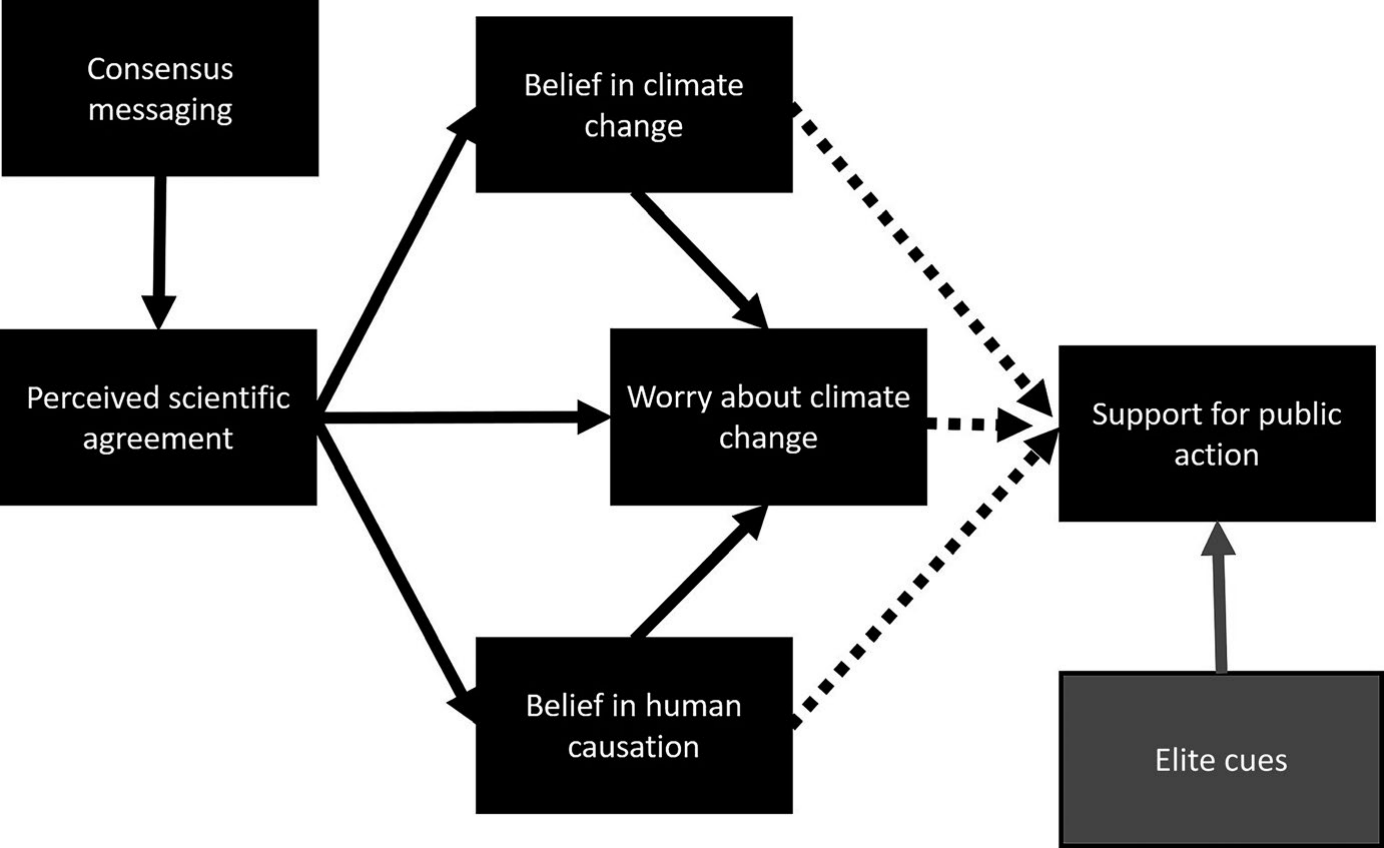
Summary of research findings and potential interventions to support belief in climate change and support for public action. Our data partially supported the gateway belief model, which was outlined by van der Linden et al. (2015) [black boxes and solid black arrows]; however, they did not indicate that increasing belief in and worry about climate change translated into support for public action for all students [black, dotted lines]. Our research suggested that using elite cues to deliver messages about climate change may be a more effective way to induce increased support for public action in more conservative audiences [gray box and arrow]. This figure was modified from van der Linden et al. (2015)

University educators are required to teach socially and politically controversial concepts to students from a wide range of political backgrounds. At the same time, university professors are increasingly more politically liberal (Gross, 2013; Gross & Fosse, 2012; Langbert, 2018) and less religious (Gross & Simmons, 2009) than the general population. Therefore, it can be challenging for professors to effectively convey information about topics that are socially and politically controversial to diverse groups of students. This study sought to evaluate the effectiveness of video‐based teaching strategies to support student acceptance of global, anthropogenic climate change. Before the video intervention, students in this study exhibited beliefs in climate change that were typically associated with their political ideology, with students who self‐identified as conservative being the most skeptical, students who identified as liberal being most in line with the consensus, and students that identified themselves as politically moderate falling between the two extremes. Notably, prior to the intervention, conservative students largely agreed that there was scientific consensus about anthropogenic climate change; however, they were unsure whether climate scientists agreed that humans are the *primary* cause of the change. Our findings provide further evidence that consensus messaging can enhance perceived scientific agreement about climate change, which can positively influence belief in and worry about climate change (van der Linden et al., 2015). Moreover, we found that if consensus messaging is delivered using elite cues, it may also promote support for public action in groups of more politically conservative students (Figure 6).

Scientific consensus and expertise are frequently cited to quell dissent. This approach, however, has yielded mixed results (Deryugina & Shurchkov, 2016; Kahan et al., 2011; Myers et al., 2015). Perceived scientific agreement can simultaneously influence belief in climate change, concern about climate change, and belief in human causation of climate change, responses that influence support for public action. This has been described as the gateway belief model (van der Linden et al., 2015), and studies have demonstrated that consensus messaging can positively influence the perception of scientific consensus (van der Linden et al., 2017). However, understanding whether consensus messaging influences support for public action is still under debate (Kahan, 2016; Linden, Leiserowitz, & Maibach, 2017). Our findings support the idea that consensus messaging can shift student understanding about scientific agreement on socioscientific concepts, resulting in beliefs that are more in line with scientific consensus. However, they do not resolve the question as to whether the resulting change translates into increased support for public action.

When teaching socially and politically controversial topics, certain groups of students may feel immediately alienated (Cook & Lewandowsky, 2016; Hart & Nisbet, 2012). Our findings support other work, suggesting that there may be no relationship, or a negative relationship, between knowledge of climate science and concern about climate change among self‐identified conservatives (Hamilton, 2011). In other words, we documented additional evidence that scientific content presented to people may not be an important factor in supporting action related to socioscientific issues that are politically controversial. Rather, our data indicate that forming a foundation of scientific consensus before presenting the detailed facts may be an effective way to address the backfire effect. Consensus messaging in our study included a description of how scientific consensus is measured and used topics that are currently not controversial (i.e., plate tectonics and gravity) to discuss how the scientific consensus around these topics compares to the consensus about anthropogenically derived climate change. This condition also explained the dubious methodology used by those who would like to overestimate doubt (i.e., counting papers that do not state a position as uncertain or doubtful of anthropogenic climate change, rather than excluding them from the analysis; Cook et al., 2016).

The inclusion of socioscientific issues in science teaching has been suggested as an effective way to increase student engagement (Tidemand & Nielsen, 2017). However, if issues are politically controversial, it is important to be careful about how information is presented. In the United States, discussions about the drivers and implications of climate change have been highly politicized, and exposure to more conservative voices that support change can be quite limited. Our work suggests that presenting students with a consistent message of concern from a diversity of voices across the political spectrum may positively influence student support for action on climate. Elite cues, rather than consensus messaging, were more effective in supporting a change in student's perception on the need to act to mitigate climate change in this study. Elite cues have been effective in combatting antivaccination sentiment (Quinn et al., 2013), a similar politically controversial but scientifically noncontroversial socioscientific issue, and we hypothesized that this effect could extend to climate change. Walker et al. (2017) determined that identity protective cognition was the phenomenon most responsible for preventing student acceptance of the scientific consensus. In other words, students may reject scientific consensus if accepting consensus on an issue was perceived to threaten their identity.

In our study, consensus messaging resulted in the greatest overall change in student belief that humans are responsible for climate change, but elite cues were the most effective in increasing student belief that humans must act to mitigate climate change. Our data suggest that using public figures with whom a viewer can identify to express the consensus position may allow for a broadening of cultural identity to include acceptance of climate change. Further, people tend to be convinced only by people they like or can identify with (Carnegie, 1936). Thus, using trusted messengers to deliver consensus messaging may establish new and positive understanding of politically controversial science. Our findings indicate that educators may want to develop teaching materials that specifically address consensus and incorporate well‐established, trusted messengers to convey the urgency with which we need to address climate change. This may be especially true in regions where students traditionally have more conservative political beliefs.

While this study revealed no significant evidence of a backfire effect, it may have lacked the power to detect this relationship. Furthermore, our study did not assess the long‐term effects of the experimental interventions. Future work should assess the efficacy of these methods in supporting long‐term changes in student responses. Our findings indicate that coupling consensus messaging with elite cues from persons generally respected by conservatives (military officials, religious figures, conservative politicians, etc.) may be an especially effective method to initiate modules on the study of climate change, especially if increasing acceptance of climate science and increasing support for action to mitigate climate change are explicit learning objectives of the instructor. Additionally, the elite cues strategy appears to have increased student support for action on climate, without increasing student worries regarding climate change. We speculate that this may be due to increasing student belief that something can be done about climate change. This coupled approach may support students in learning about and understanding the need to mitigate climate change, without increasing student stress surrounding the topic, which can be intense (Clayton et al., 2017; Ojala, 2012).

## CONFLICT OF INTEREST

None declared.

## AUTHOR CONTRIBUTIONS

**Kodiak A. Sauer:** Conceptualization (lead); data curation (lead); formal analysis (lead); investigation (equal); methodology (equal); writing–original draft (lead). **Daniel K. Capps:** Conceptualization (equal); methodology (equal); writing–review and editing (equal). **David F. Jackson:** Conceptualization (equal); formal analysis (equal); methodology (equal); writing–review and editing (equal). **Krista A. Capps:** Conceptualization (equal); data curation (equal); formal analysis (supporting); funding acquisition (equal); investigation (lead); methodology (equal); project administration (lead); resources (lead); supervision (equal); writing–original draft (equal); writing–review and editing (lead).

## Supporting information

Appendix S1

Video S1

Video S2

Video S3

## Data Availability

All of the data collected in this study are contained in the manuscript and Appendix S1.
